# Anemia and intestinal parasites in Mbya Guarani children, Misiones, Argentina

**DOI:** 10.1590/S1678-9946202466047

**Published:** 2024-08-26

**Authors:** Enrique-Jorge Deschutter, Rut-Karina Marczuk, Nestor-Guillermo Blanco, José-Manuel Ramos-Rincón

**Affiliations:** 1 Universidad Nacional de Misiones, Facultad de Ciencias Exactas, Químicas y Naturales, Departamento de Microbiología, Misiones, Posadas, Argentina; 2 Universidad Nacional de Misiones, Master de Salud Pública y Enfermedades Transmisibles, Misiones, Posadas, Argentina; 3 Ministerio de Salud Publica, Hospital SAMIC de Obera, Laboratorio de Análisis Clínicos, Misiones, Posadas, Argentina; 4 Ministerio de Salud Publica, Hospital de Campo Grande, Misiones, Posadas, Argentina; 5 Universidad Miguel Hernández de Elche, Departamento de Medicina Clínica, Alicante, Spain; 6 Hospital General Universitario Dr. Balmis, Departamento de Medicina Interna, Alicante, Spain

**Keywords:** Anemia, Intestinal parasites, Children, Mbya Guarani, Indigenous, Risk factors

## Abstract

This study aimed to assess the prevalence of anemia in children of two Guarani communities in Misiones, Argentina, and to analyze its association with socioenvironmental and parasitic factors. This cross-sectional study took place in two villages, Koen Ju and Kaa Poty, and included Mbya Guarani children aged 6 months to 14 years. A multivariable analysis was performed to evaluate the association of anemia with the presence of intestinal parasites. Altogether, 162 children were included in the study: 53.1% were boys, 32.7% had low weight-for-age, and 22.2% low height-for-age. Nearly half (46.9%, n=76) had anemia, which was mainly mild (92.1%), with a few moderate cases (7.9%). Of the 109 children who underwent testing for intestinal parasites, 89 (81.7%) had at least one, and 53 (59.5%) had more than one. The main parasite was *Blastocystis hominis* (49.5%), followed by *Entamoeba coli* (47.7%), hookworms (36.7%), and *Ascaris lumbricoides* (31.5%). In the multivariable analysis, anemia was associated with intestinal parasitosis (adjusted odds ratio [OR] 4.24, 95% confidence interval [CI] 1.08–16.5; p=0.038) and male sex (adjusted OR 2.66; 95% CI 1.08–6.47; p= 0.01). Overall, we found that both anemia and intestinal parasites are common in the pediatric population of the Guarani ethnic group. Intestinal parasites and male sex were associated with the presence of anemia.

## INTRODUCTION

Around 42 to 58 million indigenous people live in Latin America, which correspond to a range of 7.8% to 9.8% of the population^1,2^. The burden of morbidity and mortality in these communities is higher than that observed in the general population. More specifically, indigenous children have high mortality, high malnutrition and food insecurity, poor access to water, and high prevalence of diarrheal infections^3^.

The Mbya Guarani people are a branch of the Guarani ethnic group that inhabit Paraguay, southern Brazil, and the Argentinian province of Misiones. Their subsistence activities include slash-and-burn horticulture, hunting, fishing, and foraging, although the predominance of these activities has waned somewhat in recent years. Currently, in addition to temporary work on yerba mate and tobacco farms, they produce and sell artisanal crafts, which is one of their main sources of income. This allows them to acquire industrially produced food in the towns nearest to their villages. The villagers also frequently seek the healthcare system^4^. However, despite the communities access to public healthcare, the illness narratives for the most common diseases in children, such as influenza, diarrhea, parasites, among others members of these communities commonly describe patterns of resort that begin in the community—more specifically in the domestic sphere^5^ and by consulting different types of local healers, known as Karai, Kuna Karai, and Opygua^4,5^.

According to the Mbya Guarani, to create or found a new village, one must “dream” of it, give it a name, and build an Opy (prayer-house temple). Similarly, to conceive a child, it is necessary for the father to dream of them; only then will the spirit “take a seat” in the maternal womb. Then, to consider a child a “person,” that is, a full-grown member of the group, the Opygua (spiritual leader) must discover and communicate the true name of their spirit^4,5^. In essence, then, Mbya people see themselves as the fusion of a (divine) spirit/name and a (human) body. In this sense, health depends on the integration of the entities that make up the person, and their dissociation is signaled by illness or death.

Broadly speaking, anemia is a serious health problem in women of childbearing age and in children in low- and middle-income countries^3^. In children, it can be the result of multiple factors, with sociodemographic conditions playing an important role, especially in low-income countries^6-8^. However, a recent systematic review found only few Latin American studies evaluating the prevalence of anemia in indigenous children^9^, and even fewer investigating its association with intestinal parasites.

In Argentina, indigenous peoples account for approximately 2.4% of the total population^2^. In the Province of Misiones, this proportion corresponds to 0.8%, mostly comprised by the Mbya Guarani ethnic group, of whom 33.7% are aged under 15 years^10^. Most of the Mbya Guarani people live in remote villages with limited access to basic services like healthcare, and in vulnerable social conditions. Different studies have evaluated intestinal parasites in indigenous children^11-13^, but few have investigated the association of anemia with intestinal parasites^14^. This study aimed to assess the prevalence of anemia in children of two Guarani communities and to analyze its association with socioenvironmental and parasitic factors.

## MATERIALS AND METHODS

### Study design, setting, and population

**Figure 1:**
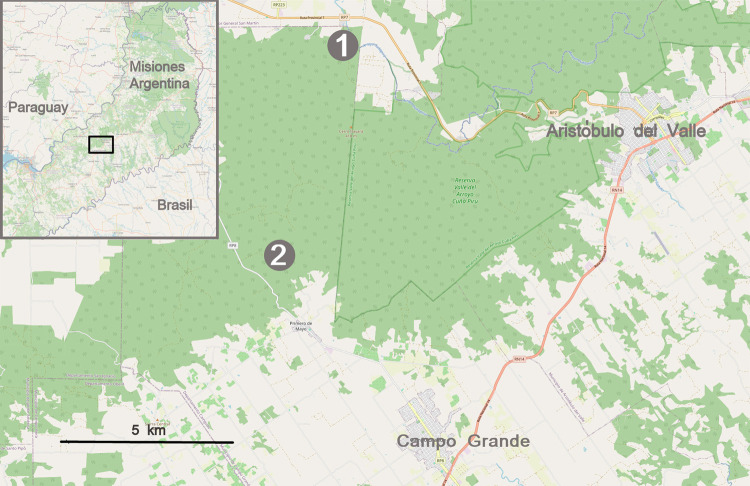
Location of villages Koen Ju (1) and Kaa Poty (2), within surrounding area in the municipality of Campo Grande (Misiones), bordering Provincial Route 7 in the Cuna Piru Valley (provincial park area).

This cross-sectional study took place in 2 of the 14 Mbya Guarani villages in the sanitary zone called Central-Uruguay in the province of Misiones (Argentina) from 15 June 2022 to 14 June 2023. Koen Ju village (with a total population of 399 in 2022) has its own primary school, water borehole, and electricity lines, while Kaa Poty village (with a total population of 50 in 2022) has no school or health center of its own, nor does it have a borehole or electricity in the homes, which are very precarious (Figure 1). Similarly, there is no proper sanitation system in the villages, nor do these undertake organized urban or rural residential planning^10^.

Inclusion criteria were: children aged 6 months to 14 years, residing in Koen Ju or Kaa Poty, without chronic disease at the time of recruitment, and with parents or guardians who agreed to participate in the study by signing informed consent. To classify children as having or not having a chronic disease, we reviewed the patient medical records available to the director of the sanitary zone that includes both villages. In the village without a health center (Kaa Poty), a primary care team makes periodic visits and records information for each child residing in the village. We excluded children with any acute illness or infection in the previous 30 days. The suspicion of acute pathologies during the execution of the study led to the clinical referral and accompaniment of the child and their guardian to health care facilities to ensure timely assistance and appropriate treatment; these patients were not included in the study. Children suffering from acute illnesses attend health services in the healthcare network of the study area according to the clinical severity of the consultation. Moreover, during the research period, no children with acute pathologies (including clinical signs and symptoms associated with anemia and/or enteroparasitosis) were identified.

### Variables and study procedure

#### Evaluation of anemia

To assess the presence of anemia, blood samples were obtained by venipuncture, and hemoglobin (Hb) levels were determined. The thresholds for defining anemia were adjusted for the child’s age: for children of 6 months to 5 years, were considered values of < 11 g/dL; for those from 5 years to 11 years, the values of < 11.5 g/dL; and for children of 12 years to 14 years, were considered < 12 g/dL. Anemia was classified as mild (Hb 10 to 10.9 g/dL), moderate (Hb 7 to 9.9 g/dL), or severe (Hb < 7 g/dL). For the diagnosis of anemia, the studies also included laboratory tests for ferritin and total red blood cell count, which were useful to validate Hb results and characterize the type of anemia. Analyses were performed in the SAMIC Hospital Obera, of the Ministry of Health of Misiones^15^.

#### Diagnosis of intestinal parasites

To test for the presence of intestinal parasites, fecal samples were collected from children whose parents provided consent. Children were considered infected with parasites if cysts, eggs, or larvae were detected in stool samples and/or on Graham’s test. Specimens were analyzed within 24 h of collection: stools were preserved in formalin (5%), and the perianal area was scraped with adhesive tape (Graham’s test) for visualization of eggs. All samples were analyzed in the Microbiology Department of the National University of Misiones (Posadas, Argentina). Each fecal sample was subjected to parasitological analysis using the method of direct coprological examination with saline and Lugol’s solution. If amoeba trophozoites were detected, temporary staining with buffered methylene blue was applied to identify the genus and species based on their nuclear characteristics. Subsequently, the formalin-ether concentration technique with formalin-saline solution was used in each sample, as described by Botero and Restrepo^16^. Trophozoites and cysts were identified based on morphology, size, number, and nuclear characteristics, according to criteria described by Alger^17^. The Baermann technique was not used to identify larvae.

#### Anthropometric variables

Body weight was measured in kg using a portable digital scale (precision 10 g), calibrated at the beginning of the day. Height was measured in centimeters using a horizontal anthropometer for infants aged 6 to 12 months and vertical anthropometer (1 mm precision) for children older than 12 months. Measurements were taken with the children barefoot and their head positioned in the Frankfort plane. To assess intra-observer concordance, each measurement was performed twice and the average of the two measurements was used for analysis. The low weight-for-age and low height-for-age index were calculated using growth charts provided by the World Health Organization, adapted with input from the Argentinean Society of Pediatrics^15^. If a child’s weight falls below the 5th percentile of the reference chart for their age and sex, they are classified as having low weight-for-age. Similarly, if a child’s height is below the 5th percentile of the reference chart for their age and sex, they are classified as having low height-for-age.

#### Socio-health surveys

Socioenvironmental data were collected by surveys, enabling the identification of conditions and behaviors that could be associated with anemia and/or parasitic infections. It was considered the use of footwear, the construction materials and the flooring of their homes, the method of waste and excreta disposal, and the availability of drinking water in the homes.

## Statistical analysis

Survey data were collected, coded, and entered into an Excel spreadsheet. All analyses were performed using SPSS program (version 25.0, IBM, Armonk, NY, USA). Statistical significance was set at p < 0.05. Categorical variables were described as frequencies (percentages) and compared using Pearson’s chi-squared test. Hemoglobin levels were described as median and interquartile range (IQR) and compared using the Mann-Whitney U test, if the variable did not follow a normal distribution. Differences in anemia (yes or no) and other categorical variables were calculated using odds ratios (ORs) with 95% confidence intervals (CI). A multivariable analysis was performed with variables that presented p values < 0.1 in bivariable analysis, except if the frequency measure was 0 in either of the compared groups.

## Ethical aspects

The project protocol was approved by the Provincial Research Ethics Committee, a department of the Government of the Province of Misiones, Argentina (protocol Nº R20-01-2024).

## RESULTS

A total of 162 children were included (147 [90.7%] in Koen Ju and 15 [9.3%] in Kaa Poty). Table 1 presents their sociodemographic, anthropometric, clinical, and parasitic characteristics. All the children lived in homes with unimproved sanitation facilities, and 89.5% had access to water from a borehole, electricity, and schooling. Just over half (53.1%) were boys, 32.7% had low weight-for-age, 22.2% low height-for-age, and 46.9% (n = 76; 95% CI 39.1–54.9%) had anemia. Of these, 70 (92.1%) had mild anemia, and 6 (7.9%) had moderate anemia. No cases of severe anemia were observed.

**Table 1:** Characteristics of participating children (N = 162)

Characteristic	N	%
Village		
Koen Ju	147	90.7
Kaa Poty	15	9.3
Age in years		
0–3	50	30.9
4–6	31	19.1
7–9	31	19.1
10–12	33	20.4
13–15	17	10.5
Sex		
Male	86	53.1
Female	76	46.9
Weight-for-age		
Normal	109	67.3
Low	53	32.7
Height-for-age		
Normal	126	77.8
Low	36	22.2
Hemoglobin (g/dL)*	11.9	(11.0–12.8)
Anemia		
Yes	76	46.9
No	86	53.1
Intestinal parasites (n = 109)		
Yes	89	81.7
No	20	18.3
Type of intestinal parasite		
*Blastocystis hominis*	54	49.5
*Entamoeba coli.*	52	47.7
Hookworms	40	36.7
*Ascaris lumbricoides*	28	31.5
*Giardia lamblia*	1	0.6
*Hymenolepis nana*	1	2.8
Pinworms	0	0.0
*Strongyloides stercoralis*	0	0.0
Polyparasitism (n = 89)		
Yes	53	59.6
No	36	41.5
Socioenvironmental conditions		
Home	162	100
Toilet	162	100
Potable water	145	89.5
Electricity	145	89.5
Schooling	145	89.5

*median and interquartile range.

Of the 109 children (67.3%) tested for intestinal parasites, 89 (81.7%) had positive results: 36 (41.5%) were infected with only one parasite, while 53 (59.5%) had more than one. All of the positive cases of anemia were identified in the village of Koen Ju (p = 0.006). The main intestinal parasite was *Blastocystis hominis* (49.5%), followed by *Entamoeba coli* (47.7%), hookworms (36.7%), and *Ascaris lumbricoides* (31.5%). No cases of *Entamoeba histolytica/dyspar* were found. Larval forms of *Strongyloides stercoralis* were not identified. Eggs of morphology and dimensions compatible with those from hookworms were not observed. All Graham’s test yielded negative. Intestinal parasites were observed in 80.2% (81/101) of children from Koen Ju and in 100% (8/8) from Kaa Poty (p = 0.164), while polyparasitism was detected in 55.6% (45/81) of the children from Koen Ju and in 100% (8/8) from Kaa Poty (p = 0.019).

Table 2 shows the median and IQR values for hemoglobin in the 109 children who contributed a stool sample for intestinal parasite testing. The children with intestinal parasites and especially with *E. coli* showed low hemoglobin values; however, there were no differences in other intestinal parasites or for cases of polyparasitism.

**Table 2:** Median and interquartile range (IQR) of hemoglobin in the 109 children providing a stool sample for intestinal parasite study by presence of intestinal parasites

	Hemoglobin levels, g/dL, median (IQR)		P

	Intestinal parasites	No parasites	
Any parasite	11.6 (10.5–12)	12.2 (11.9–13.0)	**0.006**
*Blastocystis* spp.	11.6 (10.5–12)	11.9 (11.3–12.9)	0.062
*Entamoeba coli.*	11.3 (10.3–12)	11.9 (11.6–13.0)	**0.004**
Hookworms	11.6 (10.2–12.4)	11.6 (11.0–12.0)	0.18
*Ascaris lumbricoides*	11.6 (11.0–12.6)	11.6 (10.2–12.0)	0.16
*Hymenolepis* spp.	12.0 (11.9–12.2)	11.6 (11.0–12.4)	0.37
*Giardia lamblia*	NA	NA	-
Polyparasitosis	11.6 (10.2–12.0)	11.6 (11.0–12.6)	0.33

NA = not applicable; statistically significant results (p < 0.05) in bold.

Table 3 shows the risk factors for anemia in the 109 children providing a stool sample for intestinal parasite study. No cases of anemia were observed in children who were in school and lived in homes with potable water and electricity. Intestinal parasites were detected in 90% of the children with anemia and in 73.3% of the children without (p = 0.019). The presence of *E. coli* in feces showed a borderline significant association with anemia (56.6% vs 42.9%, p = 0.070).

**Table 3:** Risk factors for anemia in the 109 children providing a stool sample for intestinal parasite study

Characteristic	Anemia				P value	Crude OR	95% CI	Adjusted OR	95% CI	P value

	No (N = 56)		Yes (N = 53)							

	n	(%)	n	(%)						
**Village**										
Koen Ju	48	(47.5)	53	(52.5)	**0.006**	NA	NA	NI	NI	NI
Kaa Poty	8	(100)	0	(0)				NI	NI	NI
**Age in years**										
0–3	26	(66.7)	13	(33.3)		Ref		Ref		
4–6	15	(66.0)	10	(40.0)	0.59	1.33	0.47–3.77	0.44	0.88–2.63	0.330
7–9	6	(50.0)	6	(50.0)	0.014	4.33	1.33–14.02	0.67	0.12–3.74	0.654
10–12	5	(27.8)	13	(72.2)	0.008	5.20	1.52–17.8	2.44	0.43–14.5	0.354
13–15	4	(50)	4	(50)	0.38	2.00	0.43–9.32	2.66	0.43–16.3	0.386
**Sex**										
Male	24	(44.4)	30	(55.6)	0.15	Ref		Ref		
Female	32	(58.2)	23	(41.8)		1.73	0.81–3.71	**2.66**	1.08–6.47	0.031
**Weight-for-age**										
Normal	39	(54.9)	32	(45.1)	0.31	Ref		NI	NI	NI
Low	17	(44.7)	21	(55.3)		1.58	0.88–3.32	NI	NI	NI
**Height-for-age**										
Normal	47	(54.7)	39	(45.3)	0.19	Ref		NI	NI	NI
Low	9	(39.1)	14	(60.9)		1.85	0.73–4.79	NI	NI	NI
**Intestinal parasites**										
Yes	41	(46.1)	48	(53.9)	**0.019**	3.51	1.17–10.4	**4.24**	1.08–16.5	0.038
No	15	(75.0)	5	(25)		Ref				
**Type of intestinal parasites**										
*Blastocystis* spp.	24	(44.4)	30	(55.6)	0.15	1.73	0.81–3.77	NI	NI	NI
*Entamoeba coli*	22	(42.3)	30	(57.7)	0.070	2.01	0.94–4.32	1.24	0.49–3.18	0.63
Hookworms	17	(42.5)	23	(57.5)	0.16	1.75	0.80–3.86	NI	NI	NI
*Ascaris lumbricoides*	16	(57.1)	12	(42.9)	0.16	0.72	0.45–1.16	NI	NI	NI
*Hymenolepis* spp.	2	(66.7)	1	(33.3)	1.0	1.92	0.16–21.8	NI	NI	NI
*Giardia lamblia*	0	(0.0)	1	(100)	0.49	NA	-	NI	NI	NI
Polyparasitism	23	(43.2)	30	(56.6)	0.54	1.30	0.55–3.06	NI	NI	NI
**Socioenvironmental conditions**										
No potable water	8	(100)	0	(0.0)	0.006	NA	NA	NI	NI	NI
No electricity	8	(100)	0	(0.0)	0.006	NA	NA	NI	NI	NI
No schooling	8	(100)	0	(0.0)	0.006	NA	NA	NI	NI	NI

CI = confidence interval; NA = not applicable; NI = not included; OR = odds ratio; statistically significant results (p < 0.05) in bold.

The multivariable model included age, sex, presence of parasites, and *Entamoeba* spp. but excluded other variables (village, availability of potable water, electricity, and schooling) that showed null values in one of the groups. Anemia was independently and significantly associated with parasitosis (adjusted OR 4.24, 95% CI 1.08–16.5, p = 0.038) and male sex (adjusted OR 2.66, 95% CI 1.08–6.47, p = 0.031). There was no association with age or *E. coli*.

## DISCUSSION

This study with Mbya Guarani children living in two villages of Misiones, Argentina, showed that nearly half of the children studied had anemia, and 8 out of 10 had intestinal parasites. Generally, the sanitary conditions of the home and the environment determined the risk of both anemia and parasitosis^6,11^.

In a recent systematic review in indigenous children in Latin America, the prevalence of anemia ranged from 4% to 100%, depending on the population, ethnicity, and age of the studied population^6^. In the Peruvian Amazon, Diaz et al. found that anemia was more frequent in indigenous compared to non-indigenous children^18^. The prevalence of intestinal parasites in our sample was 80%, similar to other studies carried out with Guarani children of Puerto Iguazu^12,13^.

No anemia was found in children from Kaa Poty, although this community had the worst socio-environmental conditions. The absence of anemia could be explained by the random selection and especially the low number of studied children in this village compared to the total number of children in the study. Additionally, there are traditional Guarani healers who operate in both villages, addressing parasitic diseases and their consequences, and this could influence the results^4,5^.

There is a known association between intestinal parasites and anemia. Several factors may be at play, including digestive loss due to parasitosis (especially hookworms), and the chronic inflammatory state due to chronic intestinal parasite infection^8,9,19^. The study by Carvalho-Costa *et al*.^9^, carried out in Brazil, also investigated *Giardia lamblia* occurrences and hemoglobin levels, but in our study *Giardia lamblia* was found only once, precluding analysis. In our study, anemia was associated with intestinal parasites in general, but not with a species in particular. It is likely that the small sample size limited the power of the statistical analysis on *E. coli* and hookworms. Another study in Misiones indicates that among the hookworms identified, the most prevalent species identified by PCR was *Necator americanus* ^20^.

The main strength of this study is the focus on an indigenous population, the Mbya Guarani, which has not been previously evaluated in the province of Misiones, as most studies with the Mbya Guarani people have been performed in the northern region of the province, around Puerto Iguazu^12-14,21^. On the other hand, limitations include the fact that the stool testing was not undertaken in all children, due to the difficulty of sample collection at the time of the study visit. Moreover, the larvae were not studied to identify *Strongyloides stercoralis*, and the narrow geographical delineation of the study area does not allow for results generalization to other pediatric populations elsewhere or to other ethnicities in Argentina. Finally, fecal matter was collected on a single day, rather than over the recommended 3–5 days, which influences the sensitivity of the diagnostic method, reducing the probability of identifying parasitic species. This design prioritized a sampling model for fecal material that allowed for quick and low-cost information on enteroparasitosis, reducing the risk of close cohabitation with fecal samples in households for several days, along with the corresponding biological exposure risk for families residing in precarious housing. However, the high prevalence results observed using this method underscore the magnitude of enteroparasitosis in the child population, with a reasonable cost-benefit ratio. Therefore, if multiple samples of fecal material considered optimal had been utilized, the prevalence results could have been even higher than those observed. Another limitation was the analysis of anemia as a dichotomous variable, rather than as continuous hemoglobin levels. Likewise, we did not study its association with anthropometric measures, as done elsewhere^8^.

## CONCLUSION

In conclusion, anemia and intestinal parasites are widespread in the pediatric population of the Mbya Guarani people in Misiones, Argentina. Anemia is associated with having any intestinal parasite and male sex. In view of these results, hygienic and sanitary measures must be implemented to protect children from environmental risk factors associated with these conditions.
